# Oxidative stress tolerance and antioxidant capacity of lactic acid bacteria as probiotic: a systematic review

**DOI:** 10.1080/19490976.2020.1801944

**Published:** 2020-08-14

**Authors:** Tao Feng, Jing Wang

**Affiliations:** a Institute of Animal Husbandry and Veterinary Medicine (IAHVM), Beijing Academy of Agriculture and Forestry Sciences (BAAFS), Beijing, China; b Sino-US Joint Laboratory of Animal Science, Beijing Academy of Agriculture and Forestry Sciences, Beijing, China

**Keywords:** Probiotic, lactic acid bacteria, oxidative stress, oxygen tolerance, antioxidant capacity, assessment method

## Abstract

Lactic acid bacteria (LAB) are the most frequently used probiotics in fermented foods and beverages and as food supplements for humans or animals, owing to their multiple beneficial features, which appear to be partially associated with their antioxidant properties. LAB can help improve food quality and flavor and prevent numerous disorders caused by oxidation in the host. In this review, we discuss the oxidative stress tolerance, the antioxidant capacity related herewith, and the underlying mechanisms and signaling pathways in probiotic LAB. In addition, we discuss appropriate methods used to evaluate the antioxidant capacity of probiotic LAB. The aim of the present review is to provide an overview of the current state of the research associated with the oxidative stress tolerance and antioxidant capacity of LAB.

## Introduction

Lactic acid bacteria (LAB) are a diverse group of gram-positive bacteria that widely exist in nature, including plants and animals. Probiotic LAB strains, especially of the genera *Lactobacillus* and *Bifidobacterium*, have various health-promoting effects. Owing to their nutritional and functional benefits to humans and animals, public interest in the application of probiotic LAB in foods and feeds has increased. However, during industrial processing and in the gastrointestinal tract, probiotic LAB are exposed to unfavorable environments, including high or low temperature, low pH, bile salts, oxygen, or limited nutrition, inducing stress.^1,2^ These stressors affect LAB survival during processing and shelf-life during storage, as well as survival, proliferation, and functionality in the gastrointestinal tract. In order to guarantee a sufficient number of viable bacteria in the final product and effective health-promoting action in the host, it is critical to isolate strains that exhibit high viability and functionality as well as high stress resistance.

Among the above stressors, oxidative stress is of critical importance as it greatly influences viability and product quality.^3^ The oxygen sensitivity of probiotic LAB is a major factor limiting their viability, although LAB are regarded aerotolerant anaerobes. Anaerobic bacteria lack the capability to synthesize an active electron transport chain,^4^ which affects their survival in aerobic environments. High oxygen levels will lead the formation of reactive oxygen species (ROS), including the superoxide anion (O_2_^–^), hydrogen peroxide (H_2_O_2_), and the highly reactive hydroxyl radical (HO·). When accumulated, ROS cause oxidative stress, which results in damage to proteins, DNA, and lipids, and even cell death.^5^ Therefore, preventing oxidative stress in LAB cells by using O_2_-tolerant LAB strains and applying adequate production and storage techniques are important to ensure high bacterial viability during storage and in the gastrointestinal tract.^2,6^

Besides a rapid and sensitive oxidative stress response, probiotic LAB exhibit substantial antioxidant activity in the host intestine and promote the production of antioxidant enzymes to help remove ROS in the host intestine and thereby alleviate oxidative damage. When host defense is weakened, various stresses can readily induce ROS production, which may result in a redox imbalance and subsequent impairment of biomolecules, which can lead to various disorders. Evidence suggests that some probiotic LAB strains can increase the activity of antioxidative enzymes or modulate and relieve circulatory oxidative stress to protect cells from oxidative stress-induced damage.^7^ Although the antioxidant properties of probiotic LAB have been confirmed *in vitro* and *in vivo*, the mechanism by which they regulate oxidative stress tolerance is not fully understood.

Various methods have been developed and used to assess the antioxidant properties of probiotic LAB. These range from methods to detect free radicals and metal ions to end-product and enzymatic assays. However, the antioxidant mechanisms of probiotic LAB are complex, and different strains use different mechanisms. Currently, there are no uniform testing standards nor a comprehensive indicator, and thus, it is impossible to compare the antioxidant capacity of different probiotic strains. Various approaches have to be combined in order to identify and characterize novel probiotic LAB for food production and as effective food and feed additives.

LAB present in fermented foods and dietary supplements for humans and animals have been recognized to have beneficial effects on health and well-being, without having any obvious adverse effects. There are numerous mechanisms through which LAB can exert these beneficial effects. Recent studies have revealed the significant antioxidant abilities of LAB both *in vivo* and *in vitro*, which may contribute to their beneficial effects and have instigated a renewed interest. In this review, we discuss the redox system of LAB and their oxidative stress tolerance, with a focus on the LAB antioxidant properties and their mode of action. In addition, we present commonly used assays and methodologies to screen LAB antioxidant capacity. Our aim was to provide a comprehensive overview of LAB oxidative stress tolerance and antioxidant capacity, and their evaluation.

## Oxidative stress tolerance

### Oxygen radical formation and toxicity in probiotic LAB

**Figure 1.**
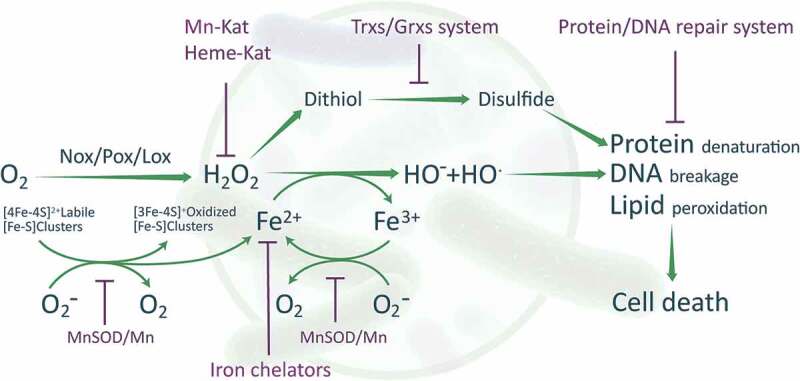
Scheme summarizing the redox system in LAB. Oxygen within a LAB cell can be consumed by several oxidases (NADH oxidases (NOX), pyruvate oxidase (POX), and lactate oxidase (LOX)) to produce H_2_O_2_. H_2_O_2_ can react with Fe^2+^ to produce free radicals, which leads to protein, DNA, and lipid damage as well as cell death. H_2_O_2_-degrading enzymes in LAB cells, such as pseudocatalase (a manganese-containing enzyme, Mn-Kat) and heme-dependent catalase (Heme-Kat) can decrease the H_2_O_2_ level. In addition, LAB can chelate iron to reduce the level of Fe^2+^. Superoxide dismutases (SODs) in LAB cells, such as MnSODs, can reduce the level of O_2_^–^, thus preventing Fe^2+^ production. The thioredoxin-thioredoxin reductase system (Trxs) and glutathione-glutaredoxin system (Grxs) in LAB cells regulate the thiol-disulfide balance and thus contribute to maintaining redox homeostasis. Other protective systems in LAB cells may contribute to the repair of damaged protein and DNA.

Oxygen is considered one of the critical factors affecting the survival of anaerobic aerotolerant probiotic bacteria. An aerobic environment may stimulate the production of toxic oxygen byproducts, such as ROS, reactive nitrogen species (RNS), and reactive sulfur species, in probiotic LAB.^5^ H_2_O_2_ produced in such a condition can react with ferrous iron (Fe^2+^) salts and produce the extremely toxic HO· through the Fenton reaction (Figure 1).^8^ HO· can damage proteins, causing a reduction in ATP and resulting in a lower energy level within the bacterial cell. HO· can also break phosphodiester bonds in DNA molecules, which leads to DNA fragmentation, and damage lipid moieties within the plasma membrane. A high steady-state concentration of O_2_^–^ can increase the release of Fe^2+^ from proteins containing iron-sulfur clusters, thus promoting the Fenton reaction (Figure 1). These radicals directly or indirectly damage proteins, DNA, and lipids, and thus eventually lead to low cell viability and cell death (Figure 1). Endogenous production of H_2_O_2_ and further reactive oxidants has been shown to be the main cause of oxidative stress in *Lactobacillus johnsonii* NCC 533 during aerobic growth.^4^ LAB lack dedicated enzymes that can eliminate HO·, but have developed other selective strategies to limit HO· formation through eliminating H_2_O_2_ and O_2_^–^^9^.

### Oxidative stress-related enzymes in probiotic LAB

#### O_2_-consuming enzymes

Some LAB have O_2_-consuming enzymes, such as NADH oxidases (NOX), pyruvate oxidase (POX), and lactate oxidase (LOX) (Figure 1).^10^ These enzymes are generally involved in the aerobic metabolism of bacteria, and especially, in microaerophilic bacteria such as LAB. NOX consumes O_2_ to form H_2_O or H_2_O_2_ in LAB.^11,12^ These O_2_-consuming enzymes are responsible for the rapid removal of O_2_ and play an important role in maintaining the intracellular redox balance. Research in NOX-defective *Streptococcus* mutants indicated that superoxide dismutase (SOD) and glutathione (GSH) reductase activities were increased to enhance oxidative stress tolerance, indicating that these enzymes have complementary actions.^13^ POX and LOX contribute to the formation of H_2_O_2_ in LAB.^14,15^ While both enzymes are expressed throughout growth, POX produces most of the H_2_O_2_ in the early and log phases, whereas LOX mainly contributes to H_2_O_2_ production in the stationary phase.^16^ Zotta and colleagues discovered that POX, but not NOX activities in *Lactobacillus plantarum* C17 were signiﬁcantly affected by temperature and oxygen.^17^

#### Antioxidant enzymes

Superoxide dismutases (SODs) are among the most important antioxidant enzymes in LAB. They dismutate O_2_^–^ and thus decrease the intracellular concentration of free metal cations and alleviate the damage caused by H_2_O_2_. Mn, Fe, and Cu are the major metal cofactors for the enzymatic function of SODs. MnSODs have been found in several LAB species (Figure 1), whereas FeSODs and Cu/ZnSODs are observed less in LAB. Further, Mn can serve as an O_2_^–^ scavenger within SOD-deficient LAB cells (e.g., *L. plantarum*).^18^ Cu forms complexes with phosphates and other proteins that exhibit O_2_^–^- and H_2_O_2_-scavenging activities.^19^ Regular H_2_O_2_-degrading enzymes such as catalases and peroxidases scarcely exist in LAB, but they have other antioxidant enzymes. A Mn-containing pseudocatalase has been discovered in *L. plantarum*,^20^ and a heme-dependent catalase has been identified in *Lactobacillus sakei*.^21^ These enzymes provide protection against H_2_O_2_ toxicity (Figure 1). Knowledge about the regulation of antioxidant enzymes in LAB is very limited. It has been demonstrated that the activity of MnSOD is dependent on the intracellular concentration of Mn^2+^,^22,23^ whereas heme-Kat activity depends on the hematin concentration.^24^

### Redox and repair systems in probiotic LAB

The thioredoxin-thioredoxin reductase and GSH-glutaredoxin systems, which maintain intracellular dithiol/disulfide homeostasis in both prokaryotic and eukaryotic cells, play an important role in the defense against oxidative stress.^25^

The thioredoxin system, comprising NADPH, thioredoxin reductase, and thioredoxin, shuttles electrons to thiol-dependent peroxidases to maintain redox homeostasis and protect probiotic bacteria from ROS and RNS damage. This system controls the thiol-disulfide balance and thus plays an essential role in DNA and protein repair by reducing ribonucleotide reductase and methionine sulfoxide reductases, and regulating the activity of numerous redox-sensitive transcription factors.^26-28^ Many LAB species have a thioredoxin-dependent reduction system (Figure 1). Overexpression of thioredoxin reductase in *L. plantarum* strain WCFS1 led to production of thioredoxin reductase, which improved the strain’s tolerance toward oxidative stress.^29^ A thioredoxin reductase mutant of *Lactobacillus casei* strain Shirota was not able to grow under aerobic conditions because it was deficient in this enzyme.^27^ Multiple thioredoxin genes have been reported in numerous bacterial species, and different levels of sensitivity to oxidative stress have been observed in strains lacking a thioredoxin gene.

Gram-negative bacteria such as *Escherichia coli* generally have a GSH-glutaredoxin-independent reduction system. Previously, it was thought that gram-positive bacteria cannot synthesize GSH and thus, do not have the GSH-glutaredoxin system, comprising NADPH, GSH, GSH reductase, and glutaredoxin, to serve as reducers.^30^ However, later studies revealed that some LAB, such as *Streptococcus agalactiae* and *Lactobacillus fermentum* E3 and E18, naturally synthesize GSH at a high level.^31,32^ Killisaar and colleagues also for the first time found that *L. fermentum* strain ME-3 has a fully functional GSH system comprising both GSH peroxidase and GSH reductase.^33^ GSH is oxidized by GSH peroxidase to a disulfide, which can be rapidly reduced back to GSH by GSH reductase in strain ME-3, suggesting that ME-3 harbors a complete GSH system (synthesis, transport, and redox recycling) that effectively protects the cells against oxidative stress. Studies on the precise physiological functions of GSH and the antioxidative role of the GSH system in gram-positive bacteria such as LAB are lacking.

### Genes associated with redox in probiotic LAB

Complete genome sequencing has been used in recent years to identify genes related to antioxidant properties and to reveal the potential mechanisms of O_2_ tolerance and antioxidant activity of probiotic LAB. Genome analysis of *Lactobacillus gasseri* AL3 and AL5 revealed genes encoding NOX and NADH peroxidases, SOD, Dps-like peroxide resistance protein, and the complete thioredoxin reductase system.^34^ Five genes encoding proteins related to free-radical scavenging and O_2_ tolerance have been revealed in *Bifidobacterium longum* LTBL16 by genome analysis, including three peroxide oxidoreductases and one NOX.^35^ Genome analysis of *Bifidobacterium animalis* subsp. *lactis* 01 showed at least eight protein-coding genes are antioxidant-related genes, and qPCR results demonstrated that genes encoding thioredoxin system and non-enzyme factors of the divalent cation transporter were upregulated under H_2_O_2_ challenge.^36^ In *B. longum* LTBL16, a gene encoding SIR2, associated with antioxidant activity, has also been identified.^37^ Genes encoding the complete GSH system, including GSH peroxidase and GSH reductase, have been identified in *L. plantarum* ZLP001.^38^ *L. plantarum* ZLP001 also harbors genes for a complete thioredoxin system, including thioredoxin, thioredoxin reductase, and thiol peroxidase. However, *L. plantarum* ZLP001 does not harbor SOD genes, implying that different LAB species encode different redox-related genes and have different redox systems.

### Strategies to increase oxidative stress tolerance in LAB

#### Coculture with starter strains

Coculture with starter strains has been demonstrated as a possible strategy to improve the survival of probiotic bacteria during fermentation. Some O_2_-depleting strains have been used as starter strains in coculture to exhaust O_2_ thus and improve the survival of O_2_-sensitive probiotic strains.^39^ However, if O_2_-sensitive probiotic species are cocultured with a strain that can produce a high level of H_2_O_2_, oxidative stress will occur and affect the viability of the cocultured microorganisms.^40^ Yeasts seem to have higher antioxidant activity than LAB. *L. plantarum* CCMA 0743 cocultured with the yeast *Torulaspora delbrueckii* CCMA 0235 exhibited increased antioxidant activity, as indicated by an α,α-diphenyl-β-picrylhydrazyl (DPPH) assay, as well as enhanced growth during fermentation, indicating the positive effect of this yeast strain on LAB proliferation and oxidative stress.^41^ Furthermore, catalase-expressing *Streptococcus thermophilus* improved the survival rate of *Lactobacillus delbrueckii* subsp. *bulgaricus* ATCC 11842 under H_2_O_2_ exposure and showed a protective effect against oxidative damage during milk fermentation.^42^

#### Addition of O_2_-consuming enzymes

The O_2_ level in a culture affects the rate of ROS production and the amounts of O_2_^–^ versus H_2_O_2_ produced. Reducing and removing the O_2_ present in the medium is beneficial to probiotic fermentation. Sasaki et al.^43^ suggested that NOX is the major O_2_-consuming enzyme of *S. thermophilus* 1131, which plays an important role in yogurt fermentation mainly through removing dissolved O_2_. Supplementation of O_2_-consuming enzymes such as glucose oxidase has been demonstrated to alleviate oxidative stress in LAB in yogurt during refrigerated storage.^44,45^ Glucose oxidase supplementation combined with a suitable packaging system with low O_2_ permeability can increase the probiotic cell density.^46^ These results indicated that the addition of O_2_-consuming enzymes may be an effective way to avoid oxidative damage, but this requires further investigation.

#### Addition of antioxidants

The addition of O_2_ scavengers or antioxidant compounds has been suggested as a possible approach to temporarily reducing the O_2_ level and improving the survival of probiotic strains. Ascorbic acid, green tea extracts, and grape extract have been verified to improve the survival of *Lactobacillus* strains through their antioxidant action.^47^ Improved survival of *Lactobacillus acidophilus* in yogurt was achieved by cysteine supplementation.^48^ Catechin supplementation significantly improved the growth of *Lactobacillus helveticus* under aerobic conditions, likely through ROS and RNS scavenging or metal ion chelation.^49,50^ Mn^2+^ is an important metal in antioxidant enzymes and when intracellularly accumulated, it can help scavenge O_2_ in *L. plantarum* during aerobic growth.^18,51^ Mn^2+^ supplementation greatly promoted the viable count of *L. plantarum* under aerobic conditions.^52^ Most LAB species cannot synthesize GSH and can only accumulate it from the medium.^31^ GSH supplementation has been found to enhance growth as well as glucose consumption, and to increase soluble protein and amino acid concentrations in *Lactobacillus reuteri* strain ATCC 23272.^53^

#### Physicochemical methods

Novel packaging materials and encapsulation technologies for improving LAB viability in an O_2_-rich environment have been evaluated. High-impact polystyrene packaging combined with O_2_-scavenging material was found to not only prevent O_2_ diffusion, but also decrease dissolved O_2_ levels during storage, suggesting it provides a more favorable environment for the LAB in yogurt.^54,55^ Encapsulation, in which small particles that contain an active agent are produced by mechanical means, has been widely used to protect probiotic strains from adverse environmental conditions. The protective effect of encapsulation on probiotic viability in an aerobic environment has also been reported. *L. acidophilus* 2409 encapsulated with calcium alginate showed significantly enhanced viability when grown aerobically in reconstituted skim milk broth.^56^ However, not all strains show improved survival under anaerobic conditions after encapsulation.^6^ Further studies are required to reveal the mechanism underlying the O_2_ toxicity-protective effects of encapsulation on probiotics.

#### Adaption and modification

Microorganisms display the ability to adapt to unfavorable environments, which has been exploited to develop strains that can survive in adverse conditions. Exposing a probiotic strain to a sublethal level of oxidative stress will induce an adaptive response and improve the resistance of the strain toward potentially higher levels of oxidative stress. This may be explained by the fact that some silent gene clusters are activated to increase the antioxidant capacity. *L. acidophilus* and *Bifidobacterium* spp. adapted to oxidative stress when dissolved O_2_ was gradually increased from 0 to 210 ppm in yogurt and could survive well for more than 35 days of storage.^57^ Genetic modification is another strategy to improve probiotic survival and oxidative stress tolerance. Heterologous expression of genes such as *KatA, Mn-Kat*, and *SodA* has been demonstrated to markedly improve the oxidative stress resistance of *Lactobacillus*.^23,58,59^ Furthermore, starter strains that can produce catalases and MnSOD and thus improve the oxidative stress resistance of LAB have been studied. Starter strain *S. thermophilus* ST5 heterologously expressing *KatE*, encoding a heme-dependent catalase, effectively eliminated H_2_O_2_ and thus improved the survival of *L. delbrueckii* subsp. bulgaricus ATCC 11842 in yogurt.^42^

## Antioxidant properties of probiotic LAB

In addition to their powerful redox systems, probiotics have strong antioxidant properties. When the body is in a state of oxidative stress, accumulated ROS will cause free-radical chain reactions through damaging biomolecules, resulting in harm to the organism. Oxidative stress is a major contributor to numerous disorders, such as cardiovascular, inflammatory, cerebrovascular, and degenerative diseases as well as aging and cancer.^60,61^ Young animals are readily exposed to oxidative damage because they lack a mature antioxidant system in their intestinal tract, leading to an imbalance in the oxidative and antioxidant systems as well as increased free radicals and malondialdehyde (MDA) and decreased antioxidant enzyme capacities.^62,63^ Multiple studies have demonstrated that probiotics, such as *Lactobacillus* and *Bifidobacterium*, possess excellent antioxidant capacity to provide a certain degree of protection against oxidative stress.^64-67^

### Modes of action of probiotic LAB antioxidants

**Figure 2.**
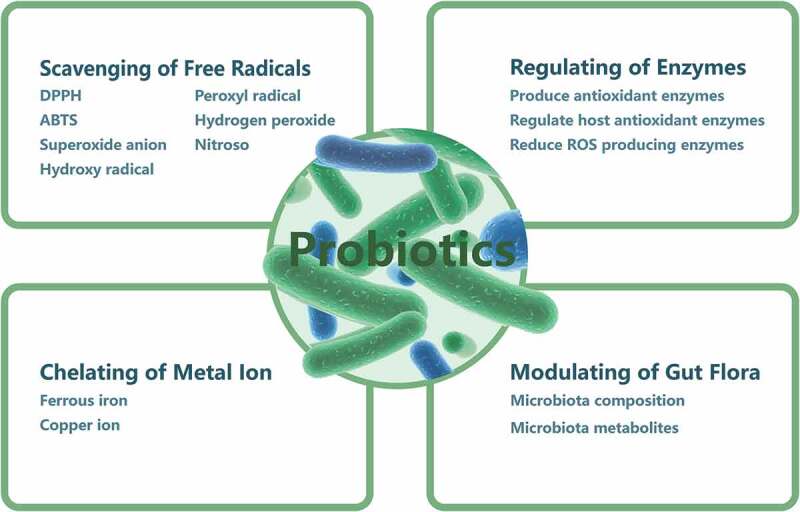
Proposed modes of action of probiotic LAB antioxidants. Probiotic LAB may exert antioxidative effects through the scavenging of free radicals, metal ion chelation, enzyme regulation, and modulation of the gut microbiota.

The mechanisms underlying the antioxidant activity of probiotic species are not completely understood; however, it has been suggested that LAB may play antioxidant roles through scavenging ROS, chelating metals, increasing antioxidant enzymes levels, and modulating the microbiota.^68,69^ The proposed modes of action of probiotic LAB antioxidants are shown in Figure 2.

#### Radical scavenging

ROS and RNS are mainly produced under exposure to stress conditions that disturb bacterial or host cell metabolism. Most LAB have systems to scavenge O_2_ free radicals through which the risk of radical accumulation during food fermentation and the damage caused by such free radicals to the host organism are lowered. Many probiotic LAB strains and their metabolites exhibit high scavenging ability toward DPPH, O_2_^–^, and H_2_O_2_ *in vitro*.^70-73^ These scavenging activities generally increase with increasing bacterial cell concentration.^71^ In one study, culture supernatant of certain strains exhibited significantly higher free-radical-scavenging activity than intact cells did,^74^ whereas in another study, intact cells showed higher scavenging activity than supernatant.^75^ This difference is probably due to differences among probiotic LAB species.

#### Metal ion chelation

In addition to the production of antioxidative enzymes, microorganisms have a non-enzymatic oxidative stress defense mechanism relying on the chelation of metal ions.^76^ Fe^2+^ and Cu^2+^ are the most prevalent and active ions generated in free-radical formation. Fe^2+^ can produce HO· through the Fenton reaction, and Cu^2+^ released from chromatin can also catalyze HO· generation. Many probiotic LAB strains have been found to possess strong chelating ability for both Fe^2+^ and Cu^2+^,^77-79^ and probiotic LAB strains show a wide range of Fe^2+^- and Cu^2+^-chelating ability, indicating that chelation capacity is strain-specific. In a study by Lin and Yen, *S. thermophilus* 821 was found to show the best ion-chelating ability among 19 LAB strains tested.^80^ In another study, *L. casei* KCTC 3260 demonstrated high chelating activity for both Fe^2+^ and Cu^2+^, at 10.6 ppm and 21.8 ppm, respectively, but did not possess detectable SOD activity.^77^ The results suggested that metal chelation may have contributed more to the antioxidative capacity of *L. casei* KCTC 3260 than SOD activation.^77^

#### Enzymatic regulation

##### Antioxidant enzyme production

As mentioned above, LAB have their own antioxidant enzymatic system. Most LAB can scavenge free radicals by producing antioxidant enzymes that dismutate free radicals to O_2_ and H_2_O_2_.^81^ SOD activity has been reported in cell-free extracts of strains belonging to *Lactococcus* and *S. thermophilus*, with *Lactococcus* exhibiting higher activity than *S. thermophilus*.^5^ The antioxidant enzymes produced by these bacteria theoretically can help prevent free radical accumulation in the host. Furthermore, LAB strains expressing high levels of SOD or catalases could be developed as a strategy in traditional food applications and new therapeutic uses. de LeBlanc and colleagues proved that engineered *Lactobacillus casei* BL23 strains producing CAT were able to prevent or decrease the severity of intestinal pathologies caused by ROS.^82^ The same team later discovered that engineered *L. casei* BL23 strains producing either CAT or SOD promoted the recovery of initial weight loss in mice with trinitrobenzenesulfonic acid-induced Crohn’s disease, increased enzymatic activities in the gut, and reduced the extent of intestinal inflammation.^83^

##### Host antioxidant enzyme regulation

LAB can induce the activity of host antioxidative enzymes, thus regulating the antioxidant system and alleviating oxidative stress. *In vitro* experiments using human Caco-2 colorectal cells have shown that *L. plantarum* Y44 can elevate catalase expression in cells damaged by 2,2′-azobis (2-methylpropionamidine) dihydrochloride.^84^ Another study using an *in vitro* model of enterocytes investigated the modulation of *L. casei* Shirota on the expression of gastro-intestinal GSH peroxidase in enterocytes.^85^ Human patients with type 2 diabetes that consumed yogurt containing *L. acidophilus* LA5 and *B. animalis* subsp. *lactis* BB12 had increased erythrocyte SOD and GSH peroxidase activities as well as higher total antioxidant status.^86^ Furthermore, increased SOD, catalase, GSH S-transferase, GSH, and GSH peroxidase activities after *Lactobacillus* supplementation have been observed not only in serum, but also in diverse tissues, including the liver, in various animals,^71,72,87^ suggesting the great antioxidant properties of LAB.

##### ROS-producing enzyme regulation

LAB can exert antioxidant action to alleviate oxidative stress damage through regulating certain ROS-producing enzymes. NOX is considered to be a major source of ROS generation. A study using combined *Lactobacillus* strains demonstrated that LAB can decrease NOX activity and *NOX-1* and *NOX-4* mRNA expression in spontaneously hypertensive rats.^88^ Cyclo-oxygenase 2 is highly associated with ROS production, and they show a reciprocal relationship.^89^ Pretreatment with *L. acidophilus* significantly downregulated the expression of cyclo-oxygenase 2 in bovine thymic macrophages challenged by the pathogenic bacterium, *Aeromonas hydrophila*.^90^ Furthermore, cytochrome P450 (CYP), the terminal oxidase in the electron transfer chain, can induce continuous ROS production.^91^ *L. casei* reportedly decreased *CYP1A1* expression in different parts of the jejunum, colon, ileum, and cecum in male rats.^92^ This downregulation of ROS-producing enzymes contributes to the antioxidant capacity of LAB.

#### Regulation of the gut microbiota

Under excessive proliferation of pathogens in the intestine, the intestinal epithelium produces and releases high levels of ROS, causing significant oxidative stress. It has been demonstrated that the gut microbiota can regulate redox signaling and affect redox homeostasis in the host.^93^ LAB supplementation can regulate the intestinal microbiota, and it has been speculated that LAB may exert their antioxidative effects partially through the reconstruction of the host intestinal microbiota composition.^94^ However, direct evidence of this is currently lacking. Dietary alteration of the gut microbiota is strongly linked to oxidative stress; in high-fat diet-fed mice treated with lipoic acid, decreased ROS and MDA and increased total antioxidant capacity showed a strong positive association with lactobacilli and a negative correlation with *E. coli* and enterococci.^95^ Supplementation of *L. johnsonii* BS15 alleviated high-fat diet-induced oxidative stress and changed the intestinal Firmicutes/Bacteroidetes ratio in mice,^96^ which suggested that modulation of the gut microbiota by LAB bacteria has the potential to improve the host redox state. However, further experiments are required to verify this speculation.

### Potential signaling pathways underlying the antioxidant actions of LAB

**Figure 3.**
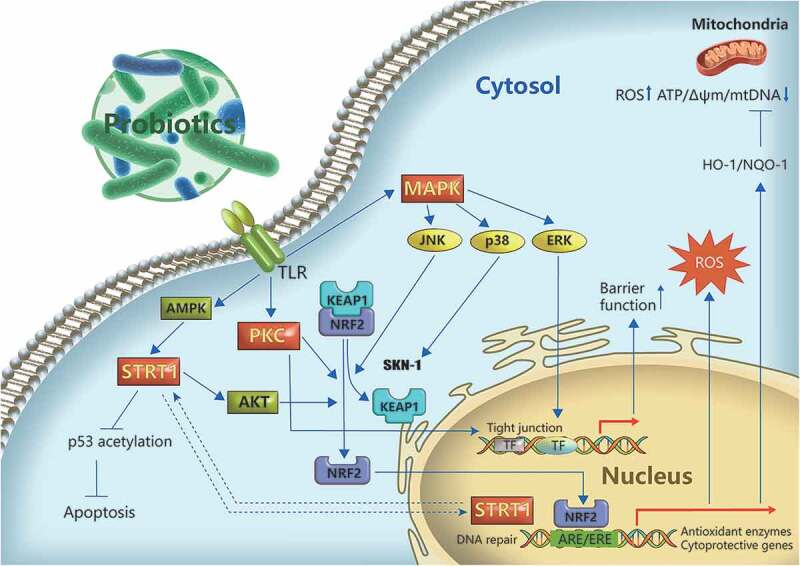
Potential regulatory pathways of LAB antioxidant action. The Nrf2/Keap1 signaling pathway plays a role in the antioxidant mechanisms of LAB in the host. In the host cells, Nrf2 is released from its cytosolic repressor Keap1 and translocates to the nucleus, where it binds to antioxidant response elements, thus enhancing the transcription of cytoprotective genes and alleviating ROS damage. LAB can activate AMPK to induce the phosphorylation and nuclear translocation of SIRT1, leading to AKT phosphorylation and Nrf2 activation. SIRT1 is required for DNA repair following H_2_O_2_-induced damage.^97^ In addition, it is involved in the protective action of LAB against p53-mediated apoptosis induced by oxidative damage.^98^ The MAPK pathway, including extracellular signal-regulated protein kinases (ERKs), c-jun N-terminal kinase (JNKs), and p38-MAPK, is involved in the regulation of antioxidant activity of LAB. JNKs and p38 are associated with the activation of Nrf2 or its ortholog, whereas ERK is related to the prevention of H_2_O_2_-induced disruption of epithelial barrier function. Protein kinase C (PKC) can also be regulated by LAB to alleviate oxidative damage. LAB can alleviate oxidative stress-induced mitochondrial dysfunction via Nrf2 signaling, strengthening the epithelial barrier function.^99^

A number of signaling pathways associated with the antioxidant mechanisms of LAB in the host, including nuclear factor erythroid-2-related factor 2 (Nrf2), silent information regulator 1 (SIRT1), mitogen-activated protein kinase (MAPK), and protein kinase C (PKC), have been identified to date (Figure 3). However, because they are generally strain-specific, likely not all signaling pathways related to LAB-induced antioxidant mechanisms have been identified, and further investigations are required.

#### Nrf2

Nrf2 is a member of the NF-E2 family of basic leucine zipper transcription factors.^100^ The Nrf2 system is well characterized as a ubiquitin-dependent signaling pathway that responds to oxidative stress.^101^ Modulation of Nrf2 signaling potentially is a novel therapeutic strategy against oxidative stress-induced complications.^102^ Under high levels of ROS, Nrf2 dissociates from its constitutive inhibitor Keap1, which contains redox-sensitive cysteine residues, and then translocates to the nucleus and binds to antioxidant response element (ARE) sequences to initiate the transcription of antioxidant and cytoprotective genes. Thus, Nrf2 activation leads to the upregulation of antioxidant and cytoprotective proteins, which play an important role in coping with oxidative stress and maintaining redox equilibrium. Nrf2 has been discovered to be one of the most important protective mechanisms against oxidant stress in probiotics.^103,104^ The involvement of Nrf2 in the activation of antioxidant cellular defenses by probiotics has been shown not only in human cells, but also in animal cell models *in vitro*.^84,85,104^ *In vivo* studies have focused mostly on different mouse models, including high cholesterol, high-fat diet, high oxidative stress, and aging models. Several studies have demonstrated that some probiotic LAB strains can activate Nrf2 signaling and upregulate downstream antioxidant enzymes including SOD, catalase, and heme oxygenase-1 in the mouse liver, thus enhancing antioxidative defense.^66,105-107^

#### SIRTs

SIRTs are an evolutionarily conserved family of NAD-dependent protein (histone/non-histone) deacetylases that play an important antioxidant role in mammals through regulating key genes and molecules integral to redox homeostasis.^97^ Recent studies have revealed that certain LAB strains harbor a *Sir2* gene.^37,108^ However, functional studies of probiotic Sir2 are rare. It has been hypothesized that LAB Sir2 plays an antioxidant role in probiotics as well as in the host. Guo et al.^37^ discovered that Sir2 exists in *B. longum* and *L. acidophilus* and seems to play a role in aerotolerance by increasing antioxidant enzyme activity. Further study showed that Sir2 in *B. longum* upregulates the expression and activity of antioxidant enzymes by deacetylating the transcription protein σ^H^. Moreover, in *in vitro* experiments using human 293 T cells, Sir2 of *B. longum* was verified to activate human MnSOD/SOD2 and catalase to reduce the cellular ROS level. Further, *B. longum* Sir2 deacetylates the forkhead transcription factor FOXO3a, which mediates antioxidant gene expression. Further studies are required to elucidate the functions of probiotic Sir2 proteins in different strains and to compare their mechanisms of antioxidant regulation. In another recent study, Bonfili and colleagues discovered that the administration of a probiotic formulation termed SLAB51 comprising bifidobacteria, lactobcilli, and *S. thermophilus* markedly alleviated oxidative stress in a mouse model of Alzheimer’s disease, and this was mediated mainly by activated SIRT1-dependent signaling as indicated by significantly increased SIRT1 activity and expression.^98^ Butyrate-producing probiotics can prevent the progression of nonalcoholic fatty liver disease involving oxidation damage through the activation of AMPK and AKT.^109^ The authors also confirmed that sodium butyrate-mediated AMPK activation induced the phosphorylation and nuclear translocation of SIRT1, leading to the phosphorylation of AKT and activation of Nrf2 *in vitro*.^109^ We speculate that probiotic strains may activate SIRT1 through this signaling pathway to induce Nrf2 expression and activation. A novel selenium-GSH-enriched probiotics strain reportedly can attenuate hepatic oxidative stress, ER stress, and inflammation caused by CCl_4_ via activating SIRT1 signaling.^110^

#### MAPKs

MAPKs, including ERKs, JNKs, and p38-MAPK, are involved in numerous signal transduction pathways. Diverse processes, including cell growth, proliferation, differentiation, inflammation, and immunization are associated with MAPK regulation. Some of these processes may be caused by oxidative stress. Several studies have demonstrated that probiotics can alleviate H_2_O_2_-induced disruption of barrier function and tight junctions in mammalian epithelial cells. The soluble proteins p40 and p75 produced by *Lactobacillus rhamnosus* GG prevent H_2_O_2_-induced disruption of human intestinal epithelial tight junctions and increase paracellular permeability, likely through the rapid activation of ERK1/2.^111^ In *Caenorhabditis elegans* fed *L. gasseri* SBT2055 (LG2055), SKN-1 (an Nrf ortholog) was activated, which induced the transcription of antioxidant genes via p38 MAPK signaling, thus enhancing the antioxidant defense response.^112^ In another study in mammalian cells, it was discovered that LG2055 treatment activated JNK, and inhibition of JNK activation suppressed Nrf-2 ARE signaling activation and the protective effect of LG2055 against oxidative stress.^113^ This indicated that LG2055 may activate Nrf2-ARE signaling through JNK activation, thus strengthening the antioxidant defense in mammalian cells.^113^

#### PKC

PKC is a family of protein kinases that control protein function through the phosphorylation of hydroxyl groups of serine and threonine residues. Evidence suggests that PKC is a target of redox modification because it contains unique structural features that are susceptible to oxidative modification,^114,115^ and this activity may be involved in various pathways that regulate cell growth and barrier function as well as stress responses.^116-118^ PKC-ζ improves microtubule and gut barrier integrity by preventing iNOS production caused by oxidants, and therefore, is considered as an endogenous stabilizer to prevent oxidative damage.^119,120^ Zhou et al.^121^ reported that administration of *L. plantarum* improved intestinal barrier function and oxidative stress in obstructive jaundice rats by enhancing the PKC pathway in terms of expression and activity. Furthermore, H_2_O_2_-induced epithelial barrier disruption can be ameliorated by the soluble proteins p40 and p75 produced by *L. rhamnosus* GG through a PKC- and MAPK-dependent mechanism.^111^

### Antioxidant molecules produced by probiotic LAB

#### Exopolysaccharides (EPS)

EPS are group of carbohydrate polymers that play important roles in biofilm formation and cell adhesion, and are produced also by probiotics.^122,123^ EPS have various beneficial physiological functions in humans and animals, including the regulation of intestinal barrier function^124^ and the immune response.^125,126^ Moreover, EPS from *Lactobacillus* have been shown to exert excellent antioxidant activity *in vitro* and *in vivo*.^127^ *In vitro*, purified EPS improved the aerobic growth of *Leuconostoc mesenteroides* by ~10-fold.^128^ The aerobic growth of O_2_-sensitive probiotics was enhanced by EPS through the relief of O_2_ stress, which was achieved through the extrusion of dissolved O_2_ in biofilms and aggregations^129^ or in aqueous environments.^128^ EPS from different probiotics exhibit prominent and concentration-dependent free-radical-scavenging and metal-chelating activities.^73,130,131^ The protective effect of EPS from *Lactobacillus* against host oxidative stress have been evaluated in different cell lines, such as Caco-2 and PC12.^132,133^ EPS from *L. plantarum* LP6 and *L. plantarum* C88 exhibit antioxidant effects by improving cell viability and downregulating oxidative stress biomarkers.^132,133^ *In vivo* studies have shown that EPS from probiotics increase antioxidant enzyme activities and decrease end products of redox processes in the liver and in serum, indicating their excellent antioxidant effects.^134,135^ EPS may also have indirect effects via regulating the microbiota composition or shielding bacterial cell-wall surfaces.^125,136^ It is challenging to completely elucidate the molecular mechanism underlying the antioxidant action of EPS because EPS are highly diverse in terms of structure and physicochemical properties.^137^ Moreover, EPS production is affected by various conditions, including temperature, pH, and O_2_ strength.^138,139^

#### Carotenoids

Carotenoids, which are widespread in nature, have well-known antioxidant properties. Carotenoids are common in pigmented bacteria and their terpenoids possess 30, 40, or 50 carbons. C_50_ carotenoids are restricted to certain gram-positive bacteria, C_40_ are commonly present in photoautotrophic bacteria, and C_30_ are found in some unrelated genera and species.^140^ Some probiotic bacteria produce carotenoids, and this is likely associated with their antioxidant activity. *Lactobacillus pentosus* KCCP11226 harbors C_30_ carotenoid biosynthetic genes (*crtM* and *crtN*), shows high carotenoid production and survival under oxidative stress, and is considered a functional probiotic.^141^ Notably, aerobic growth conditions, while slowing down growth, signiﬁcantly induced carotenoid production in *Enterococcus gilvus,*^142^ which indicated that aerobic culture conditions may contribute to conferring oxidative stress tolerance in carotenoid-producing LAB. Further research revealed that aerobic growth conditions not only affected *crtN* and *crtM* expression, but also the biosynthesis of the carotenoid precursor isoprenoid via mevalonate in *E. gilvus*.^143^ Transcriptome analysis of oxidative stress-response genes in *E. gilvus* corroborated that the regulation of isoprenoid biosynthetic genes is one of the potential mechanisms of the carotenoid-based oxidative stress response in LAB.^144^

#### Ferulic acid (FA)

FA is natural phenolic acid that is abundantly presented in many types of foods, such as cereals, fruits, and coffee. FA is a potent antioxidant that can eliminate free radicals through a neutralization reaction.^145^ Some probiotic bacteria produce feruloyl esterase (FE), which hydrolyzes and releases FA from its bound state^146,147^ and thus exerts health-beneficial antioxidant properties. Based on qualitative precipitation and quantitative HPLC assays, *L. fermentum* NCIMB 5221 was found to produce the most active FE among several bacteria tested,^148^ and antioxidant capacity tests verified its significant antioxidant activity. An *in vivo* study revealed that *L. fermentum* CRL1446 supplementation enhanced the production and bioavailability of FA in mice through increasing the activity of FE, exerting an antioxidant effect and improving the host oxidative status.^149^ FE activity was affected in a time- and dose-dependent manner, and optimal intestinal FE activity was observed on day 7 after supplementation of 10^7^ CRL1446 cells per day. It can be inferred that probiotic strains might secrete FE enzymes into the intestine or regulate intestinal microbiota to directly stimulate FE activity. Further studies confirmed that FA-producing probiotics can induce metabolic changes and exert beneficial effects on the host metabolic state.^150^ This potent beneficial activity of FA can be explained by the modulation of certain metabolites and inflammatory markers linked to antioxidant activity.^150,151^

#### Histamine

Histamine produced by *Lactobacillus* species has been demonstrated to have immunoregulatory functions, comprising pro- as well as anti-inflammatory effects.^152^ Recent studies have revealed that histamine also plays a role in the antioxidant potential of lactobacilli. Histamine inhibits the generation of superoxide radicals by activated macrophages,^153^ and histamine dihydrochloride-treated human leucocytes showed increased catalase activity and decreased SOD activity.^154^ Histamine was found to be produced by *L. reuteri* strains, including *L. reuteri* E and *L. reuteri* KO5, under both aerobic and anaerobic conditions.^154^ The concentration of histamine increased along with an increase in lactobacilli cells, and the maximum concentration was reached not earlier than after 48 h.^154^ Cell culture supernatants of lactobacilli that produced histamine modulated the enzymatic activities of SOD and catalase.^154^ However, the presence of an adequate amount of precursor, i.e., biogenic amine, is required for LAB to produce histamine; thus, the effect of histamine produced by LAB on antioxidant capacity remains to be thoroughly studied.

## Methods to assess the oxidative stress tolerance and antioxidative properties of probiotic LAB

Various methodologies have been used to assess the antioxidative properties of probiotics, and these can be classified based on the target of detection, mechanism of action, and measure adopted. Most of the methods used to assay common antioxidants can be applied to probiotics. Some procedures adopt biochemical approaches to test the radicals generated (external or cellular), some use specific techniques to detect the end products, and others involve eukaryotic-cell and animal testing. While the testing methods used in probiotics research are extremely versatile, it is important to choose the most appropriate assay to assess the antioxidant properties of probiotic strains. As the various antioxidative assays have their own characteristics, it is difficult to compare results among studies using different assays.^155^ Furthermore, various methods are often combined because no single method can provide unequivocal results.^156^

### Assays of oxidative stress tolerance

#### O_2_ tolerance

The relative bacterial growth ratio (RBGR) method to evaluate the O_2_ tolerance of probiotics was originally developed by Kikuchi and Suziki^157^ and later optimized by Talwalkar and colleagues.^3^ The RBGR is calculated as the ratio of the absorbance (representing growth) of an aerobically shaken culture to that of an anaerobically shaken culture. The O_2_ tolerance of probiotic LAB strains can be measured quantitatively using the RBGR index, and an RBGR value close to 1 indicates good O_2_ tolerance. The RBGR method is a simple and easy approach that can be used for high-throughput screening of probiotic bacteria. Li and colleagues used this method to evaluate 10 strains of bifidobacteria from various sources in several O_2_ concentrations and to select strains exhibiting high O_2_ tolerance.^158^

#### Resistance to H_2_O_2_

Another method to evaluate the O_2_ resistance of probiotics is to evaluate their viability in H_2_O_2_. H_2_O_2_ is a weak oxidant that can permeate the cell membrane and subsequently cause damage.^69^ While probiotics are generally resistant to H_2_O_2_ as they produce H_2_O_2_ in the intestinal tract as an antimicrobial compound, this assay is commonly used to evaluate the viability of probiotics in an anaerobic environment.^159,160^ It is worth mentioning that H_2_O_2_ can be degraded by catalase; however, most *Lactobacillus* strains do not exhibit catalase activity. Some studies have suggested that the expression of *trxB1* or *uvrA* may contribute to the survival of lactobacilli in the presence of H_2_O_2_.^29,161^ However, the precise mechanism requires further exploration.

### Assays of antioxidant properties

#### Assays based on radical production or scavenging

Free radical detection is widely used to evaluate the antioxidant activity of various antioxidants.^156^ Most antioxidant evaluation methods based on reactive species can be applied to probiotics to evaluate their antioxidant capacity.^69,162^ Radical production and scavenging systems are the most straightforward methods, and most of them use biochemical methods without the requirement for eukaryotic cells. These biochemical assays are mostly based on fluorescence or chromophore reactions, and thus are straightforward and cheap. Radical production and scavenging systems are normally used to detect the antioxidant ability of probiotic strains *in vitro*, but they have also been used to evaluate radical production after probiotic treatment or supplementation *in vivo*.^5,163^ Further, these systems can be applied to intact cells as well as cell-free extracts and cell lysates or their metabolic products.^72,75,164,165^ Reactive species tests have also been applied to evaluate the antioxidant capacity of LAB-fermented foods, especially, milk.^166^ The methods used to assess the antioxidative capacity of probiotic LAB based on the detection of reactive species are summarized in Table 1. Most studies combine assays to evaluate the antioxidant capacity of probiotic strains.^72,181^

**Table 1.** Methods for the screening of the antioxidant capacity of LAB based on radical production or scavenging.

Target radical	Method or solution	Principle	Probiotic LAB strains studied	Reference
ABTS	Trolox equivalent antioxidant capacity assay	Measures the ability of antioxidants to scavenge the stable radical cation 2,2′-azinobis (3-ethylbenzothiazoline-6-sulfonic acid), which is intensely colored.	7 *Bifidobacterium*, 11 *Lactobacillus*, 6 *Lactococcus*, and 10 *S. thermophilus*	^5^
DPPH	DPPH radical solution	Antioxidants can reduce the free, stable, and purple-colored 2,2-diphenyl-1-picrylhydrazyl radical to the yellow-colored diphenylpicrylhydrazine.	*L. plantarum* FC225	^167^
			*L. plantarum* Y44	^160^
			*L. helveticus* KLDS1.8701	^168^
Superoxide radical	Fluorescent dihydroethidium (DHE)	O_2_^–^ production is measured based on reaction with the fluorescent dye dihydroethidium.	Probiotic formulation VSL#3	^169^
	Nitro blue tetrazolium (NBT)	The scavenging activity of O_2_^–^ is analyzed based on the color reaction of NBT, NADH, and phenazine methosulfate.	*Lactococcus lactis* ssp. lactis CLFP 100, *Leuconostoc mesenteroides* CLFP 196, and *L. sakei* CLFP 202	^170^
			*L. reuteri* SHA101 and *Lactobacillus vaginalis* SHA110	^171^
	Pyrogallol autoxidation	Pyrogallol can autoxidize in solutions to produce O_2_^–^. Antioxidants can affect the production of O_2_^–^ by pyrogallol autoxidation.	*L. plantarum* FC225	^167^
			*Enterococcus faecium* BDU7	^172^
			*L. plantarum* L.P2	^79^
Hydroxyl radical	1,10-phenanthroline/FeSO_4_	HO· scavenging activity is analyzed based on the reaction of 1,10-phenanthroline, FeSO_4_, and H_2_O_2_, producing a colored product.	*L. plantarum* FC225	^167^
			*L. plantarum* LP6	^132^
			*L. plantarum* Y44	^160^
			*E. faecium* WEFA23	^173^
	Brilliant green	HO· levels in the Fenton system are indirectly detected based on the fact that HO· can make brilliant green fade.	11 *Lactobacillus* strains	^174^
			*Lactobacillus paraplantarum* D-3	^175^
	Salicylic acid	HO· scavenging activity is analyzed based on the principle that salicylic acid can be used as trapping reagent of HO·.	*L. acidophilus* LA5 and *B. animalis* subsp. *lactis* BB12	^176^
			*L. mesenteroides* S81	^177^
Peroxyl radicals	Oxygen radical absorbance capacity (ORAC) assay	The fluorescence intensity of fluorescent molecules such as β-phycoerythrin decreases over time under reproducible and constant flux of peroxyl radicals.	*Lactobacillus* spp.	^164^
			*L. fermentum* LF31	^178^
Hydrogen peroxide	Horseradish peroxidase (HRP)	HRP mediates the oxidation of phenol red by H_2_O_2_, which results in the formation of a compound that absorbs at 610 nm.	*Lactobacillus* spp.	^179^
Nitroso	α-naphthylamine	The scavenging activity of nitroso is determined based on the color reaction of sulfanilic acid and α-naphthylamine.	*Leuconostoc citreum* B-2	^180^

#### Assays based on the dynamics or end products of redox processes

Lipid peroxidation is the best-studied biologically relevant free-radical chain reaction. Although lipid peroxidation generally occurs late in the oxidative damage process, after damage of proteins and DNA,^182^ lipid peroxidation detection is among the assays the most commonly used to assess the dynamics of isolated redox processes.^162^ The extent of lipid oxidation can be determined by measuring the loss of unsaturated fatty acids or the amount of peroxidation products.^182^ Several assays are available to measure lipid peroxidation; however, as with other free-radical assays, no single method can accurately account for the entire process. Thiobarbituric acid (TBA) and diene-conjugate assays are relatively simple, but nonspecific assays. In probiotic research, the TBA assay is one of the methods the most commonly used to evaluate antioxidant capacity. The suppression of lipid peroxidation by several *L. acidophilus* and *B. longum* strains was measured using the TBA method, and the result was confirmed by detecting the lipid peroxidation product-scavenging ability.^183^ Noureen and colleagues measured the levels of lipid peroxidation inhibition of 16 LAB strains from different sources using the TBA assay to compare their antioxidant potential.^72^ They found that intact cells (47–82.38%) and culture supernatants (41–74.34%) showed higher lipid peroxidation inhibition activity than did cell lysates (10–48.92%).^72^

End products of oxidative stress are regularly used to evaluate the oxidative damage caused by ROS. Oxidized products of proteins (nitrate tyrosine, protein carbonyls), nucleic acid bases (8-hydroxy-2-deoxyguanosine), carbohydrates (glycated products), and lipids (malondialdehyde, isoprostanes, lipoproteins) can all be used as biomarkers of oxidative damage.^184^ These molecules can be easily detected using specific techniques, such as ELISA, HPLC-UV, HPLC/UPLC-MS/MS, and GC-MS. Lipid peroxidation products are the most commonly detected. MDA is the breakdown product of major chain reactions leading to the oxidation of polyunsaturated fatty acids, and it causes severe oxidative stress as a mutagenic and carcinogenic reactive substance. It serves as a reliable marker of oxidative stress-mediated lipid peroxidation in biological systems and foods. MDA assays have been widely used to study the effects of probiotic LAB in mammalian cells as well as serum, the liver, and colonic mucosa.^71,74,80,185^ Isoprostanes, another specific ROS-induced lipid peroxidation product, have served as an oxidative status marker to evaluate the effect of dietary *L. fermentum* ME-3 supplementation in humans.^186^ Lipoproteins as another lipid oxidation product have been detected in the plasma and liver of cholesterol-fed rats to evaluate the antioxidant effect of *Lactobacillus* GG.^187^ Furthermore, oxidation products, protein carbonyls, and 8-hydroxy-2-deoxyguanosine have also been used as indicators in mice or piglet serum to evaluate the antioxidant response after LAB administration.^188,189^

#### Assays based on the reducing power

The reducing power of antioxidants can be measured through redox reactions with transition metal ions, such as Fe (ferric reducing antioxidant potential, FRAP) and Cu (cupric reducing antioxidant capacity, CUPRAC).^162^ FRAP evaluates the antioxidant ability based on the reduction of ferric 2,4,6-tripyridyl-s-triazine complex [Fe^3+^−(TPTZ)2]^3+^ to [Fe^2+^−(TPTZ)2]^2+^ depending on the available reducing species, along with a color change from yellow to blue in acidic condition.^69^ The FRAP test has been used to evaluate the antioxidant capacity of *L. plantarum* Y44 in Caco-2 cells damaged with 2,2′-azobis (2-methylpropionamidine) dihydrochloride *in vitro*, which revealed that *L. plantarum* Y44 exerted antioxidative effects in a dose-dependent manner.^84^ The total antioxidant capacity in the livers of hyperlipidemic rats significantly increased after *L. casei* supplementation as indicated by a FRAP assay.^87^ FRAP assays have been also used to compare the antioxidant capacities of fresh skimmed, pasteurized, and UHT milks before and after fermentation with several lactobacilli combined or not with the yeast *Saccharomyces boulardii*.^190^

### Other methodologies

#### Assays based on DNA damage

Most types of environmental stress damage host biomolecules, including DNA. Cells with increased DNA damage display increased DNA migration from the nucleus toward the anode as indicated by photomicrography; thus, the extent of DNA migration in single-cell microgel electrophoresis under alkaline conditions can be used to estimate DNA damage.^191^ The prevention of oxidative stress-induced DNA damage by *Lactobacillus* has been evaluated in different mammalian cells (Caco-2 cells, HCT 116 cells, HT-29 cells, and lymphocytes) using a single-cell gel electrophoresis assay (Comet assay), which was developed based on DNA migration.^192-195^ The DNA-protective capacity of probiotic LAB can also be detected by molecular biology techniques to estimate oxidative stress protection.^196^ Nowak and colleagues used the DNA repair enzymes endonuclease III and formamidopyrimidine-DNA glycosylase to evaluate the antioxidant capacity of *Lactobacillus* toward H_2_O_2_ and several human carcinogens based on the recognition of oxidized DNA bases by DNA repair enzymes.^192^ Furthermore, the DNA-protective capacity of a LAB-fermented honey-based kefir beverage was assessed using a pPICZα C plasmid DNA, based on the supercoiled DNA cleavage level, indicating its DNA-protective effect against HO·-induced damage.^196^

#### Assays based on the biosensors

A biosensor is defined as an analytical device that combines a biological recognition component with a physicochemical detector and is used for the detection of a substance through generating a measurable signal.^197,198^ Microbial biosensors integrating microorganism(s) with a transducer have been also developed.^199^ In recent years, this approach has been adopted to evaluate the antioxidant properties of probiotics *in vitro*. Bacterial biosensors have been genetically modified to express the luxCDABE operon, encoding bioluminescence and luciferase, under the control of oxidation reaction-related gene promoters, such as *SoxS* or *RecA* promoters. The antioxidant activity of cell-free culture supernatant of lactobacilli has been evaluated using the biosensor strain *E. coli* MG1655, which harbors plasmids encoding the luminescent biosensors pSoxS-lux and pKatG-lux, which are inducible by O_2_^–^ and H_2_O_2_, respectively.^200^ Eukaryotic cells have also been used to construct biosensing systems to assess the antioxidant capacity of LAB. Ge et al.^201^ immobilized RAW 264.7 macrophage cells using a one-step acidified MnO_2_-modified gold electrode and then encapsulated the cells in a 3D cell-culture system. This biosensor can be used to determine the flux of H_2_O_2_ released from RAW 264.7 macrophages after treatment with LAB to indirectly evaluate the antioxidant capacity of the latter. This biosensor platform demonstrates the potential for rapid, sensitive, and quantitative screening of the antioxidant properties of LAB.

## Conclusion

In the last several decades, the oxidative stress tolerance and antioxidant capacity of probiotic LAB, as well as their health-promoting roles have been extensively investigated. The antioxidative property of probiotic strains has been confirmed in numerous studies, and the application of LAB in oxidative stress-related diseases has been investigated. Probiotic LAB strains have powerful redox systems associated with antioxidative enzymes and oxidative damage repair systems, which contribute to their O_2_ tolerance and functional roles. Probiotic LAB exhibit remarkable antioxidant capacity mainly by scavenging free radicals, chelating pro-oxidative metal ions, regulating relevant enzymes, and modulating the gut microbiota. As such, they can contribute to prolonging the shelf lives of food products and promoting health and redox equilibrium in the body. The antioxidant mechanisms of probiotic LAB involve a complex signaling network, mainly Nrf2 redox signaling. There are numerous assays for the antioxidative properties of probiotic LAB, based on different mechanisms and methodologies. However, many questions remain unanswered today. The principle and strategies of O_2_ resistance in probiotic LAB are not completely understood and require further studies. The mechanisms of antioxidant action of probiotic LAB have not been fully elucidated, and thorough pathway studies are needed to uncover the mode of action and achieve targeted use. Furthermore, the lack of a standardized and calibrated antioxidant capacity detection procedure and evaluation criteria makes it impossible to compare results among studies, and detection strategies and comparative methods need to be further investigated. However, we are hopeful that these questions will be answered in future studies.
